# The Spectrum of Sex and Gender

**DOI:** 10.1007/s10508-025-03331-y

**Published:** 2025-10-18

**Authors:** Linda Arrighi

**Affiliations:** Department of Psychology, Durham University, South Road, Durham, DH1 3LE UK

Sex and gender are intrinsic and ubiquitous aspects of the human experience. Right after we make our entrance in the world, and often even when we are still in the womb, our existence is tied to a specific sex and/or gender—signaled by the color of the cake at a gender reveal party, the color of the decorations at a baby shower, or the joyous calls of “It’s a boy!” or “It’s a girl!” Although sex and gender have been shown to overlap (e.g., sex and gender likely correspond in individuals who identify as cisgender; Veldhuis et al., 2024) and intersect (e.g., both sex and gender contribute to performance in a few specific cognitive tasks; Cartier et al., 2024; Kheloui et al., 2023) in certain contexts, there are some important distinctions to be made between the two. Sex is related to someone’s anatomy and physiology and is assigned at birth upon clinical observation of (primarily) external genital organs (King, 2022) and is, for this reason, also referred to as (birth-)assigned sex or sex registered at birth. The sex someone can be assigned at birth is among one of the following three options: male, female, and intersex but there are also other ways in which sex can be determined biologically beyond clinical observation at birth, for example through examining chromosomes or hormone levels (King, 2022). The concept of gender identity, also referred to as affirmed gender, instead relates to how someone conceptualizes and expresses their own gender (Morgenroth & Ryan, 2018). It has been shown that someone’s own concept of their gender emerges during early childhood through interactions with family and peers, and is generally stable from 3 to 5 years of age onwards (Steensma et al., 2013). Gender is both an important aspect of the self as well as an extremely salient aspect of our gendered society—it determines how we present ourselves to those around us and how we are perceived and treated.

The fact that sex and gender do not correspond in all individuals makes it critical to distinguish them. For instance, sex and gender likely do not correspond in individuals who identify as transgender, and intersex individuals’ gender identity may be subjective and dependent on one’s own traits and experiences (Thorne et al., 2019; Veldhuis et al., 2024). The assumption that the concepts of sex and gender are interchangeable is rooted in biological determinism and essentialist ideas (van Anders, 2024). These ideas argue that our gender is handed to us the moment we are born and strictly the same as our assigned sex—completely excluding the possibility that our sociocultural environment can have any effect on our concept of self. These essentialist ideas are also often accompanied by persistent gender stereotypes, rooted in the assumption that people of different sexes and genders are biologically different in their traits and talents (Brescoll et al., 2013).

A recent example of biological determinism and essentialism comes from Trump’s January 2025 inauguration speech and executive order. As Zucker (2025) noted, Trump, in his inauguration speech, first declared that “There are only two sexes: male and female” and later that “There are only two genders: male and female” (CNN, 2025). This indicated a perfect conflation of sex and gender as well as an implicit assumption that gender is biologically determined. In the executive order, shortly after, it was stated that “The sex of a human, female or male, is determined genetically at conception” (Department of Health & Human Services, 2025), which is inaccurate in multiple ways. First, the assumption that sex is binary is driven by outdated ideas and scientifically inaccurate, as will be discussed in more detail in the latter part of this commentary. The statement is also untrue based on the timing of sexual differentiation during fetal development, which is initiated at around 8–9 weeks of gestation rather than at conception (McCarthy, 2010; McCarthy et al., 2018).

The executive order also stated that “A person’s sex is unchangeable and determined by objective biology” (Department of Health & Human Services, 2025) and included definitions of the terms male, female, man, and woman which clearly demonstrated an assumption that sex and gender (1) are one and the same and (2) reflect essential and biological characteristics we are born with. These ideas perpetuate sex/gender stereotypes and can be extremely damaging, hurtful, and exclusionary of individuals whose sex and gender do not correspond, or are outside the sex/gender binary (Howansky et al., 2022).

Another reason that makes it critical to distinguish sex and gender is that numerous aspects of health and disease intersect with either sex or gender, and sometimes with both (Gesensway, 2001; Gillies & McArthur, 2010; McCarthy et al., 2012; Wierenga et al., 2023). For instance, bodily changes through the teenage years (e.g., menstruation and associated conditions, such as endometriosis) are sex-specific (Adler et al., 2024). The prevalence, presentation, and treatment of certain conditions are also sex-specific. For example, as the symptoms associated with myocardial infarction and the efficacy of certain treatments differ between males and females (Ferry et al., 2019), physicians need to consider their patients’ sex to ensure appropriate and timely intervention. From just these few examples, it is glaringly obvious how accurately and consistently measuring someone’s sex—separate from gender—is necessary to timely diagnosis and sometimes lifesaving in health contexts.

In certain clinical contexts, it may be useful to measure someone’s gender alongside their sex. For example, the prevalence and presentation of mental health and neurodivergent conditions differs across people of different sexes and genders. These differences are likely the result of the combination of sex-specific neurological susceptibility and gender-specific adaptations to the sociocultural environment (Wierenga et al., 2023), including persistent stereotypes related to how people of different sexes and genders are expected to behave. For example, it is assumed that masking in autistic females and women and, more generally, sex/gender differences in autism presentation exist because there are two distinct autism phenotypes (male and female). Differences in presentation are instead likely attributable to gender stereotypes and the assumption that there are distinct types of autism may be detrimental to timely diagnosis and is not reflected in the lived experiences of autistic people (Pearson & Rose, 2021, 2023). In these instances, knowing an individual’s sex and gender is important for accurate diagnosis and treatment (where appropriate). Asking for people’s sex and gender is easy to implement and quick to administer, but still not the standard protocol in medical settings, where sex and gender are often conflated and terminology such as male/man and female/woman are used interchangeably, as noted by McCartney and Bewley (2025). Sex and gender have been at times conflated in research and academic settings as well (Cameron & Stinson, 2019).

This journal’s Editor seems to share the frustration with the conflation of sex and gender (Zucker, 2025). Notably, sparked by the previously discussed inaugural speech and executive order by Donald Trump (CNN, 2025; Department of Health & Human Services, 2025), the call that this commentary is responding to specifically mentioned two questions: (1) How many sexes are there? and (2) How many genders are there? Historically, the concepts of sex and gender have been socially constructed, understood, and accepted as binary, i.e., our traditional cultural notion of the presence of meaningful differences between men and women has in turn shaped our interpretation of biological evidence as supporting rather than refuting sexual dimorphism (Hyde et al., 2019; Morgenroth & Ryan, 2018). Recent research has instead challenged the binary notions of sex and gender—highlighting how they oversimplify reality, are exclusionary, and have remarkable economic costs for our society.

Specifically for sex, recent reports in the fields of toxicology and neuroendocrinology have recommended moving away from conceptualizing sex as a binary, as assigning sex out of a few options negates the large extent of individual variability (King, 2022; Smiley, 2024). These individual differences have been observed at the genetic, molecular, hormonal, anatomical, physiological, psychological, and behavioral level within what we would clinically and societally categorize as belonging to the same sex category, as well as across categories (Bhattacharyya, 2024; Hyde et al., 2019; Smiley, 2024). Critically, the extensive individual variability makes it extremely difficult and sometimes inaccurate to fit all individuals within either of the three neat and practical categories of male, female, and intersex (King, 2022). For example, most females have two X chromosomes and most males an X and a Y chromosome, but there are around 40 combinations, also defined as intersex conditions or differences in sex development, that diverge from this, and sometimes chromosomes are incomplete or inactivated (Jones, 2018). This can lead to extremely variable presentations across individuals (Carpenter, 2018; Jones, 2018; Moran, 2020).

While scientific advancement has highlighted the extent and abundance of sex variability, the definition of sex is still debated and part of the scientific community recognizes and accepts the gametic definition of sex, i.e., sex is determined by the gametes present in the individual, sperm, or eggs (Parker et al., 1972). Because only two gametes exist, this definition of sex is necessarily binary. Within this definition, any divergence in terms of chromosomes and/or gametes is considered an anomaly rather than evidence for the existence of more than two sexes (e.g., King, 1945). However, the gametic definition of sex implicitly reduces individuals to their capability to reproduce and conceptualizes evolutionary success as being exclusively reliant on reproduction. In contrast with this notion, research has shown that certain individuals (including intersex individuals) may adapt and pass on their genes through kin selection (e.g., helping own relatives to raise children) rather than direct reproduction—thus diversifying how evolution may take place (Hamilton, 1964; Sun, 2024). Additionally, the gametic definition of sex is exclusionary, leads to stigma and alienation of intersex individuals, and overlooks the variability and overlap in physiology and presentation across individuals who would be categorized as the same sex, as well as across sexes.

For example, it has been shown that conditions that are relatively common such as polycystic ovary syndrome (it is estimated that up to one in five women have this condition, with similar estimates [16.6%; Lauritsen et al., 2014] when using the Rotterdam criteria; Teede et al., 2010) are associated with higher testosterone levels than would generally be expected in females (Bhattacharyya, 2024; Schattmann & Sherwin, 2007). Alongside this, secondary sex characteristics such as body hair may be more aligned with a typical male presentation. Another example comes from research measuring hormone levels suggesting that there is a moderate degree of overlap in males’ and females’ hormone profiles (e.g., estradiol and progesterone), such that the only binary in terms of meaningful differences in hormonal profiles would comprise “pregnant females” and “everyone else” (Hyde et al., 2019; Tulchinsky et al., 1972). Due to large individual variability, hormone levels are not and should not be used to categorize individuals as more or less male (or female) than others (Hotine, 2021).

Overall, the research presented suggests that historically endorsed binary classifications of sex (e.g., stating that all individuals are either male or female) are, at a minimum, biologically inaccurate (Bhattacharyya, 2024). While the addition of an intersex category demonstrates an attempt at inclusion, it others individuals who do not meet the implicit binary expectations of sex, i.e., how one should look like to fit the label of male or female. The categorization of individuals as male, female, and intersex, although widely recognized as appropriate, is, at least partly, a biased interpretation of anatomy and physiology influenced by longstanding binary views of sex and gender (Hyde et al., 2019; Morgenroth & Ryan, 2018). To ensure scientific accuracy and true inclusion, it may be more appropriate to define sex as a spectrum which entails several traits (King, 2022), from the genetic to the behavioral, with the more typical male and female presentations at either end.

The newer concept of gender has been, similar to that of sex, socially constructed as a binary (Morgenroth & Ryan, 2018), including only the identities of man and woman and associating specific characteristics and talents to each. However, reports have shown that there are countless more gender identities and extensive variability in gender expression, gender fluidity, and choice of pronouns across individuals (Thorne et al., 2019; Yarbrough, 2018). For individuals whose gender generally matches their assigned sex (i.e., likely to identify as cisgender), the experience and expression of their own sex and gender may be straightforward and untroubled. In contrast, individuals whose gender differs from their assigned sex and/or from binary categories of man and woman (i.e., likely to identify as transgender) may experience distress about this mismatch as well as society’s expectations and rules around gender identity and expression (Cooper et al., 2022; Ristori & Steensma, 2016).

Indeed, there is a body of literature dedicated to understanding the experience of gender dysphoria (Ristori & Steensma, 2016; Steensma et al., 2013), including in neurodivergent individuals, who may deal with multiple intersecting challenges and find it especially difficult to subscribe to societal expectations of gender (Cooper et al., 2022; Kourti & MacLeod, 2019). Across this literature, it has been reported that the lack of societal acceptance is a significant source of distress for gender dysphoric and transgender individuals (Cooper et al., 2022). To ensure representation and true inclusion, gender may also be best categorized as a spectrum, with the more typical identities of man and woman (or, if preferred, masculinity and femininity) at either end.

Categorizing both sex and gender on a spectrum rather than through discrete categories stems from similar concerns, including (1) a need for scientific accuracy and (2) a desire to include and welcome individual variability. However, categorizing sex as a spectrum and gender as a spectrum may not be equally well received. Recent years have seen increased recognition and understanding of gender diversity across the general population (Hammack & Wignall, 2023), making the concept of gender as a spectrum relatively more digestible and acceptable than that of sex as a spectrum. This is because a good portion of the general population, especially in Western countries, would agree that while gender can be a spectrum, sex is untouchably binary (Bhattacharyya, 2024). As discussed above, categorizing sex as a spectrum is more scientifically accurate and hence closer to biological reality. Notably, a common essentialist argument that is often lumped together with the conflation of sex and gender is that conceptualizing sex as a spectrum denies biological reality. Essentialists may use statements such as “there are only two sexes,” “you cannot change your sex,” and “but you are really a male (or female)” which, while not completely inaccurate or always intended to be hurtful, may invalidate the experiences and the identity of transgender individuals (e.g., Hotine, 2021).

In this vein, holding onto binary and deterministic sex has been, especially recently, the reason for divisive and often rage-fueled debates in the media, online, and in politics about, among others, the definitions of sex, gender, man, woman, male, and female. A recent example has culminated in the Supreme Court ruling in the UK declaring that a “woman” is an individual who is assigned female at birth, and not an individual who identifies as a “woman” on their Gender Recognition Certificates (Supreme Court of the United Kingdom, 2025). Although maybe not directly implied by this statement, this suggests that someone who identifies as a “woman” but was not assigned female at birth is not a real woman—which can be an incredibly hurtful and invalidating statement for transgender women. The recent increased attention and debates on transgender rights has led to increased rates of discrimination and violence against transgender people in Europe, with a recent study showing that 58% of the 19,669 transgender individuals surveyed reported discrimination and 40% reported being attacked at least once in the previous year (Evje et al., 2024). Transgender individuals were shown to experience more discrimination and violence than the 118,543 cisgender lesbian, gay, and bisexual (LGB) individuals surveyed, 40% of whose reported discrimination and 25% reported violence in the previous year. Notably, the rates of discrimination and violence experienced by transgender individuals were higher than those experienced by cisgender LGB individuals irrespective of the countries' rankings of LGBTQIA+ rights (Evje et al., 2024). These findings hint at a worrying disconnect between the growing acceptance of sex and gender diversity across the scientific community and the notions and stereotypes commonly held by the general population.

Beyond the damage to individuals, holding onto outdated binary concepts of sex and gender has tangible and measurable costs across multiple sectors. For example, assuming sex is binary in healthcare settings can lead to increased mortality, higher treatment costs, delayed diagnoses or misdiagnoses, and lower quality of life (e.g., All Party Parliamentary Group on Endometriosis, 2020; Kersey, 2024; Wu et al., 2018). As research and care protocols are designed and carried out based on the binary of male and female, with male often being the default, billions are spent annually and research funding is wasted due to (1) the longer time needed to reach an appropriate diagnosis for conditions that differently or disproportionally affect people of different sexes/genders, and (2) the lack of effective treatment options for conditions that differently or disproportionally affect people of different sexes/genders (Kersey, 2024; World Economic Forum, 2024). Additionally, higher mortality and lower quality of life are preventable consequences of intersex and transgender people delaying or avoiding medical care due to discrimination and/or dismissive interactions with healthcare professionals (Baugher et al., 2024; Kcomt et al., 2020; Seelman et al., 2017). Sex/gender health gaps lead to an enormous financial burden—it is estimated that addressing sex/gender gaps in cardiovascular disease care protocols could add about $28 billion to the USA economy each year by 2040, while also providing at least 1.6 million additional years of quality life (Kersey, 2024). More generally, conducting research that can help narrow the sex/gender health gap has the potential to increase the global economy by over $1 trillion each year by 2040 (World Economic Forum, 2024).

These preventable costs are particularly tangible and troubling with the recent censorship campaign to research investigating “gender” and “diversity” in the USA (Graham et al., 2025) and the Supreme Court ruling in the UK declaring that sex is binary and biologically determined, and that protected characteristics are defined through assigned sex and not Gender Recognition Certificates (Supreme Court of the United Kingdom, 2025). This redirection of funding efforts and negative attitudes toward sex-/gender-related research is likely to worsen the financial burden of under-researching conditions that disproportionately or differently affect females, women, intersex, and transgender individuals.

In workplaces and education, assuming sex and gender are binary and implementing binarily gendered policies (e.g., uniforms, dress codes, and restrooms) may lead to discrimination and alienation of transgender and intersex employees and students, who may resign or be less motivated to get good grades (Kosciw et al., 2021). News outlets in the UK have been documenting the increased harassment following the Supreme Court ruling suggesting that restroom access is determined by an individual’s assigned sex rather than their affirmed gender (Supreme Court of the United Kingdom, 2025). An example is a report of a cisgender woman being challenged in women’s restrooms due to other users suspecting her of being transgender because of her ambiguous presentation (Brooks, 2025).

Individuals resigning from jobs or withdrawing from their studies due to discrimination and harassment, as well as a loss of human potential, has remarkable financial costings related to companies being involved in costly sex-/gender-related discrimination lawsuits and replacing employees who resign (U.S. Equal Employment Opportunity Commission, n.d.). Obtaining lower grades in school and resigning from jobs due to discrimination may, in turn, result in lower lifetime earnings and increased reliance on assistance programs and benefits systems—something which can have notable public costs (Badgett et al., 2019). From just these few examples, it becomes clear that there are serious financial, legal, and human costs that can be prevented by moving away from binary concepts of sex/gender.

In sum, sex and gender are concepts with important distinctions as well as intersections, especially in health and disease, but the simple answer to the questions (1) How many sexes are there? and (2) How many genders are there? is that it is inappropriate to answer either of these questions with a number because both concepts are better conceptualized as a spectrum comprising of several traits, rather than a series of discrete and countable categories. This conceptualization is more scientifically accurate than traditional binary classifications, can reduce the extensive operational and financial costs caused by holding onto rigid sex/gender binaries, and may be a catalyst for acceptance and inclusion of individuals of all sexes and genders.
